# Engineering, Expression in Transgenic Plants and Characterisation of E559, a Rabies Virus-Neutralising Monoclonal Antibody

**DOI:** 10.1093/infdis/jiu085

**Published:** 2014-02-07

**Authors:** Craig J. van Dolleweerd, Audrey Y-H. Teh, Ashley C. Banyard, Leonard Both, Hester C. T. Lotter-Stark, Tsepo Tsekoa, Baby Phahladira, Wonderful Shumba, Ereck Chakauya, Claude T. Sabeta, Clemens Gruber, Anthony R. Fooks, Rachel K. Chikwamba, Julian K-C. Ma

**Affiliations:** 1 Research Centre for Infection and Immunity, Division of Clinical Sciences, St George's University of London, United Kingdom; 2 Council for Scientific and Industrial Research (CSIR), Biosciences, Pretoria, South Africa; 3 Agricultural Research Council-Onderstepoort Veterinary Institute (ARC-OVI), OIE Rabies Reference Laboratory, Onderstepoort, Pretoria, South Africa; 4 Wildlife Zoonoses and Vector Borne Disease Research Group, Animal Health and Veterinary Laboratories Agency (AHVLA), Surrey, United Kingdom; 5 Department of Chemistry, University of Natural Resources and Life Sciences, Vienna, Austria

**Keywords:** rabies, post-exposure prophylaxis, RIG, monoclonal antibody, *Nicotiana tabacum*

## Abstract

Rabies post-exposure prophylaxis (PEP) currently comprises administration of rabies vaccine together with rabies immunoglobulin (RIG) of either equine or human origin. In the developing world, RIG preparations are expensive, often in short supply, and of variable efficacy. Therefore, we are seeking to develop a monoclonal antibody cocktail to replace RIG. Here, we describe the cloning, engineering and production in plants of a candidate monoclonal antibody (E559) for inclusion in such a cocktail. The murine constant domains of E559 were replaced with human IgG1κ constant domains and the resulting chimeric mouse-human genes were cloned into plant expression vectors for stable nuclear transformation of *Nicotiana tabacum*. The plant-expressed, chimeric antibody was purified and biochemically characterized, was demonstrated to neutralize rabies virus in a fluorescent antibody virus neutralization assay, and conferred protection in a hamster challenge model.

Rabies is a zoonotic disease caused by rabies virus (RABV), the type member of the *Lyssavirus* genus, and is responsible for >55 000 deaths per annum [1] largely in the developing world [2–4], where transmission usually occurs following the bite of an infected dog. If left untreated, the virus progressively infects surrounding neurons and propagates in the central nervous system leading, almost invariably, to death. The disease can be prevented by post-exposure prophylaxis (PEP), which consists of administration of inactivated RABV vaccine together with passive antibody therapy [5–7]. In passive antibody therapy, rabies immunoglobulin (RIG), derived either from immunized human (HRIG) or equine (ERIG) sources [8–11], is infiltrated into the wound site.

However, in the developing world, these serum-derived antibodies often suffer from drawbacks including limited availability, batch-to-batch variation, high cost, contamination with blood-borne adventitious agents, and/or risk of adverse reactions [12]; for these reasons, the World Health Organization (WHO) encourages the development and evaluation of alternative biologics for RIG replacement [13]. One such alternative is offered by monoclonal antibodies (mAbs) that are capable of neutralizing a wide range of RABV isolates [12, 14–18]. Rabies neutralizing antibodies are directed against the viral glycoprotein, and several studies have demonstrated that rabies-specific mAbs can protect rodents after RABV challenge [18–23].

However, given the unique epitope specificity of individual mAbs compared to polyclonal antiserum, any mAb-based product designed to replace RIG would ideally comprise a defined cocktail of RABV-neutralizing mAbs that would provide coverage against a broad range of RABV isolates, minimize the potential for viral escape and have a potency comparable to that of RIG. The low production costs, ability of plants to assemble and modify multimeric proteins such as mAbs, and ease of scalability make plants a viable platform for production of mAbs to replace RIG [24, 25].

Several groups have characterized RABV-neutralizing mAbs [14, 17, 25–30], and the World Health Organization Rabies Collaborating Centers (WHO RCCs) identified 5 murine mAbs [15], with 4 (E559.9.14, M727-5-1, M777-16-3 and 1112-1) recognizing antigenic site II of the glycoprotein and 1 (62-71-3) recognizing antigenic site I [31].

Amongst the mAbs identified by the WHO RCCs that recognize antigenic site II, E559 exhibited the broadest virus neutralization spectrum and greatest potency [15, 32] and therefore represents an important candidate mAb for inclusion in a RIG-replacement cocktail. In this study, we describe the cloning and sequences of the murine E559 antibody heavy and light chains, engineering of a chimeric mouse-human version of E559, expression in tobacco, and characterization of the purified, tobacco-derived, chimeric mAb in terms of in vitro virus neutralization and in vivo protection.

## MATERIALS AND METHODS

### Cell Lines, Viruses and Plasmids

Hybridoma cell line E559.9.14 [15, 32], expressing murine IgG1κ mAb E559, was kindly provided by Dr Thomas Müller (WHO Collaborating Centre for Rabies Surveillance and Research, Friedrich-Loeffler-Institute, Germany). Cells were cultured at 37°C, under a 5% CO_2_ atmosphere in CD hybridoma medium (Life Technologies) supplemented with 10% (v/v) heat-inactivated, fetal bovine serum (Life Technologies) and 2 mM L-glutamine (Sigma, UK). For mAb production, the cells were adapted to serum-free conditions.

Lyssavirus strains used included challenge virus standard (CVS) [ATCC VR-959], derived from the original Pasteur virus [33] and animal-derived isolates, as well as RV61, isolated from a person bitten by a dog.

The pL32 and pTRAk.2 plasmids used for plant transformation are described in detail in the online Supplementary Materials.

*Agrobacterium tumefaciens* strain LBA4404 was purchased from Invitrogen UK. *A. tumefaciens* strain GV3101::pMP90RK was obtained from the Deutsche Sammlung von Mikroorganismen und Zellkulturen GmbH (Leibniz Institute, Germany).

### Cloning of Full-length Murine E559 IgG

Total RNA from hybridoma cell line E559.9.14 was isolated from 1 × 10^6^ cells using the RNeasy Mini kit (Qiagen). First strand complementary DNA (cDNA) was prepared using the Omniscript RT kit (Qiagen) with oligo-(dT)_15_ as the primer.

Using the first strand cDNA as template, the murine γ1 heavy chain gene was amplified using primers FR1γ and 932 (see online Supplementary Table 1 for a description of oligonucleotide primers). The murine κ light chain gene was amplified using primers FR1κ and 933. The murine γ1 heavy chain and κ light chain amplicons were digested with *Xho*I and *Eco*RI and ligated into binary vector pL32 restricted with the same enzymes.

### Cloning of Chimeric Mouse-human E559 IgG

The cloning of the chimeric (mouse-human) heavy (χE559H) and light (χE559L) chain genes, and the codon-optimised versions of these genes, is described in detail in the online Supplementary Materials.

### Generation and Screening of Transgenic *Nicotiana tabacum* Plants

The generation of transgenic plants is described in the online Supplementary Materials. For screening of plants by Western blotting and enzyme-linked immunosorbent assay (ELISA), leaf discs were excised from leaves using the lid of a 1.5 mL Eppendorf tube as a punch. Leaf discs were homogenized using a plastic pestle in 300 µL of PBS, centrifuged at 20 000 × *g* for 3 minutes, and the supernatant collected for analysis. Total soluble protein content of the supernatant was measured using the bicinchoninic acid (BCA) protein assay kit (Pierce, UK).

### Purification of mAbs

For purification of the hybridoma-derived mAb (E559^Hyb^), hybridoma E559.9.14 cells were grown for 7 days in serum-free conditions, centrifuged (1000 × *g*, 10 minutes, 4°C) to pellet the cells, and the supernatant filtered (0.2 µm) and applied to an anti-mouse IgG1 (heavy chain specific)-agarose (Sigma, UK) affinity column.

The plant-expressed chimeric antibody (χE559^P^) was purified using Protein A/G agarose as described elsewhere [34]. In the case of the plant-expressed murine E559 (muE559^P^), an anti-mouse IgG1 (heavy chain specific)-agarose (Sigma, UK) affinity column was used instead.

Column fractions were analyzed on Coomassie stained SDS-PAGE gels. Fractions containing the antibody were pooled, dialyzed against phosphate-buffered saline (PBS), and stored in aliquots at −20°C. Dialyzed material was analyzed by ELISA and SDS-PAGE to determine the concentration, purity, and integrity of the mAb.

Samples destined for animal challenge studies were purified using MabSelect SuRe protein A chromatography on a 5 mL HiTrap column (GE Healthcare). In addition to affinity purification, samples were further purified using Capto Q (GE Healthcare) in flow through mode and polished using ceramic hydroxyapatite (CHT; BioRad Laboratories). All chromatography steps were conducted on an Akta Avant 150 operated via Unicorn 6.0 software.

Antibody concentrations were determined using a sandwich ELISA, by capturing samples with a heavy-chain specific reagent and detection with a light chain specific reagent. Commercially available human IgG1κ (The Binding Site, UK) and mouse IgG1κ (Sigma, UK) were used as concentration standards.

### Deglycosylation Using PNGaseF

The deglycosylation protocol using PNGaseF is described in detail in the online Supplementary Materials.

### Glycan Analysis of the Plant-derived mAb E559

A glycoproteomic analysis was undertaken by in-gel digestion of *S*-carbamidomethylated sample and analysis by reverse-phase electrospray ionization mass spectrometry (RP-ESI-MS), as described elsewhere [35]. Tandem MS results were also subjected to Mascot MS/MS ion search (Matrix Science Ltd, London, UK; http://www.matrixscience.com).

### Enzyme-Linked Immunosorbent Assay

ELISA for detection of antibody heavy or light chains is described in detail in the online Supplementary Materials.

### SDS-PAGE and Western Blotting

Polyacrylamide gel electrophoresis (PAGE) and Western blotting protocols are described in detail in the online Supplementary Materials.

### Modified Fluorescent Antibody Virus Neutralization (mFAVN) Assay

Live virus experiments were performed using a modified form of the fluorescent antibody virus neutralization (FAVN) assay described for CVS-11 [36, 37] and described in more detail in the online Supplementary Materials. OIE positive (OIE+) and OIE negative (OIE−) reference sera were included as controls. Virus was considered neutralized if the neutralization titer was >0.5 IU/mL [36].

### Hamster Challenge Studies

Four groups of Syrian hamsters were included in the experiment. The challenge and treatment schedule was as follows: Group 1 (uninfected control) comprised 4 hamsters that did not receive any viral inoculum or biologics treatment. Group 2 (4 animals) and groups 3 and 4 (each comprising 9 animals) were all inoculated with 50 µL of 1 × 10^6^ TCID50/mL of a RABV laboratory strain, Challenge Virus Standard CVS (at day 0) intramuscularly and treated subsequently (at day 1) with either PBS (group 2), or with 22.5 IU/kg of either undiluted commercial HRIG (Rabigam [150 IU/mL], National Bioproducts Institute, Pinetown, South Africa) (group 3) or χE559^P^ mAb (group 4). Biologics (groups 3 and 4) and PBS (group 2) were administered in the gastrocnemius muscle in 50 µL volumes to simulate passive immunization in PEP treatment. No rabies vaccine was administered. The hamsters were observed twice daily over 28 days for any symptoms associated with RABV infection. Brain tissues were collected from animals to confirm rabies virus infection for all those hamsters that succumbed during the observation period and assessed for the presence of lyssavirus antigen using the fluorescent antibody test (FAT) [38]. All hamsters surviving for up to 28 days post-infection were killed with isoflurane and tested for rabies as described above. The animal experimental protocols, animal caging and care, as well as end point for the experiments were approved by the Animal Ethics Committee for the use of living vertebrates for research, diagnostic procedures, and product development (Agricultural Research Council-Onderstepoort Veterinary Institute, South Africa).

## RESULTS

### Cloning of Antibody Heavy and Light Chain Genes From Hybridoma E559.9.14

**Figure 1.**
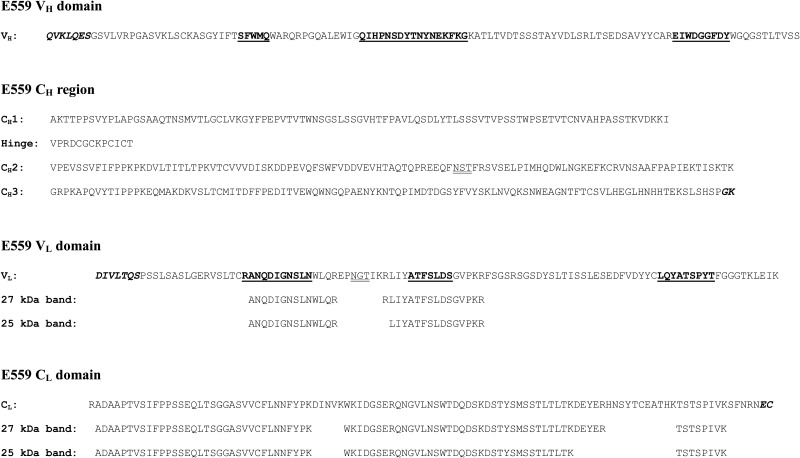
Sequences and mass spectrometry analysis of E559. Deduced amino acid sequences of the heavy chain variable domain (V_H_), the heavy chain constant region domains (C_H_1, Hinge, C_H_2, and C_H_3), the light chain variable domain (V_L_), and the light chain constant domain (C_L_) of E559. Complementarity determining regions (CDRs), as defined by Kabat et al [39], are highlighted in bold and underlined. Amino acids encoded by the primers used for amplification are shown in bold italics. Potential N-linked glycosylation sites are double-underlined. Peptides identified by mass spectrometry analysis of the 25 kDa and 27 kDa isoforms of the E559^Hyb^ light chain are shown aligned below the corresponding V_L_ and C_L_ sequences (see text).

The murine immunoglobulin γ1 heavy and κ light chain genes expressed by the E559.9.14 hybridoma were amplified by polymerase chain reaction, using first strand cDNA as template. The deduced amino acid sequences of the E559 heavy and light chain genes are presented in Figure 1. Highlighted are important features, such as the complementarity determining regions [39] and the presence of potential N-linked glycosylation sites within the C_H_2 domain and the light chain V_L_ domain.

### Analysis of Hybridoma-derived E559

**Figure 2.**
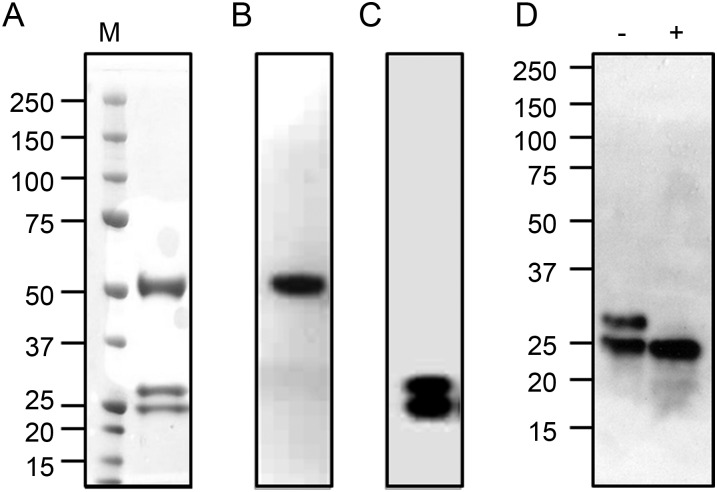
Analysis of hybridoma-derived E559. Hybridoma-derived E559 (E559^Hyb^) was purified by affinity chromatography and analyzed by SDS-PAGE under reducing conditions, followed by staining with Coomassie Brilliant Blue (*A*) or blotted to nitrocellulose and probed with HRP-labeled antisera specific for murine γ1 heavy chains (*B*) or murine κ light chains (*C*). Purified E559^Hyb^ was also treated with PNGaseF, and proteins were separated by SDS-PAGE under reducing conditions, blotted to nitrocellulose and probed with HRP-labeled light chain-specific antiserum (*D*). Lane M: molecular weight standards; (−): untreated E559^Hyb^; (+): PNGaseF-treated E559^Hyb^. Abbreviation: HRP, horseradish peroxidase.

Analysis of the purified murine hybridoma-derived E559 (E559^Hyb^) by SDS-PAGE under reducing conditions, followed by Coomassie staining, showed the presence of 3 bands with molecular weights of 50 kDa, 27 kDa and 25 kDa (Figure 2*A*). Western blotting confirmed previous findings [15] that the 50 kDa band corresponded to the heavy chain (Figure 2*B*), and that the 2 lower molecular weight bands were murine light chains (Figure 2*C*). The 2 lower molecular weight bands were excised from the gel, treated with trypsin, and analyzed by LC-MS. The panel of peptides generated from each band were nearly identical and in accord with the sequence deduced from the cloned light chain gene (see Figure 1), indicating that these 2 bands are murine κ light chain isoforms. Minor differences in the identified peptides are likely due to differences in the extent of trypsin digestion between the 2 samples. The identification of a potential N-glycosylation site within the V_L_ domain of the light chain (Figure 1) suggested that the difference between the light chain isoforms might be due to the presence of N-linked glycans. E559^Hyb^ was deglycosylated by treatment with PNGaseF. Blotting under reducing conditions shows that after treatment with PNGaseF, the 27 kDa band is lost, leaving only a single band at 25 kDa (Figure 2*D*), providing evidence that the 27 kDa species is a glycosylated form of the light chain and the 25 kDa band is the aglycosylated species.

### Characterization of Plant-derived E559

Murine and chimeric (mouse-human) heavy and light chain genes were cloned into the binary vector pL32 and transformed into *Agrobacterium tumefaciens*. Co-cultivation of *Nicotiana tabacum* leaf discs with *A. tumefaciens* strains harboring the recombinant pL32 binary vectors was used to generate transgenic tobacco lines expressing murine heavy (pL32-muE559H), chimeric heavy (pL32-χE559H), murine light (pL32-muE559L), or chimeric light (pL32-χE559L) chains. Several independent plants lines derived from each transformation were screened by ELISA to identify transgenic plants expressing each antibody chain.

**Figure 3.**
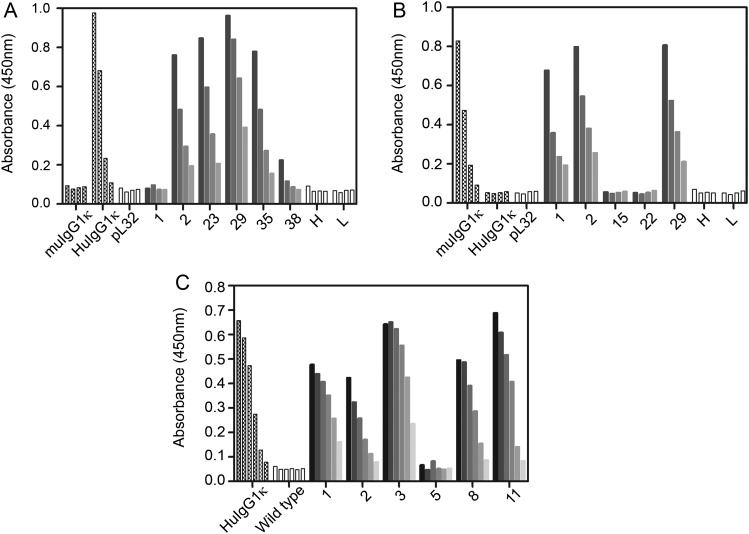
ELISA analysis of transgenic plants expressing fully assembled E559 monoclonal antibodies. Leaf discs from selected independent plant lines expressing either (*A*) chimeric E559 (pL32-χE559), (*B*) murine E559 (pL32-muE559), or (*C*) codon-optimised, chimeric E559 (pTRAk-χE559), were extracted in PBS and loaded onto ELISA plates coated with either sheep anti-human IgG1 (panels *A* and *C*) or sheep anti-mouse IgG1 (panel *B*) antisera. Bound antibodies were detected with HRP-labeled antibodies specific for either human κ light chains (*A* and *C*) or murine κ light chains (*B*). Control samples were: isotype-matched, commercially available human IgG1κ (HuIgG1κ) or mouse IgG1κ (muIgG1κ) antibodies; samples from transgenic plant lines expressing only the heavy or light chains of the chimeric E559 (H or L, respectively in panel *A*); samples from transgenic plant lines expressing only the heavy or light chains of the murine E559 (H or L, respectively, in panel *B*); a plant line transformed with nonrecombinant binary vector (pL32); and a wild-type (nontransgenic) plant. For panels *A* and *B*, plant samples were serially diluted 2-fold, whereas the isotype-matched controls were serially diluted 5-fold. In panel *C*, all samples and controls were serially diluted 4-fold. Abbreviations: ELISA, enzyme-linked immunosorbent assay; PBS, phosphate-buffered saline.

Sexual crossing was used to produce plants lines expressing the fully assembled chimeric E559 (pL32-χE559) or fully assembled murine E559 (pL32-muE559). Plants were analyzed by ELISA for antibody assembly and expression levels. The results from a selected set of plants provide evidence that both the chimeric (Figure 3*A*) and murine (Figure 3*B*) antibodies are assembled. Control plants expressing only the heavy chain (pL32-χE559H or pL32-muE559H) or the light chain (pL32-χE559L or pL32-muE559L) did not produce any signal above that of the nonrecombinant pL32 control. In sum, 5 of the 6 plants shown in Figure 3*A* expressed the chimeric antibody, whereas 3 of the 5 plants shown in Figure 3*B* expressed the murine antibody.

Using a commercially available human IgG1κ as an ELISA standard, the best expression level of the chimeric E559 (χE559^P^) was calculated as 1.8 mg/kg of fresh leaf weight (0.04% of total soluble protein), whereas the best yield achieved from the plant-derived, murine E559 (muE559^P^) was 1.2 mg/kg of fresh leaf weight (0.03% of total soluble protein).

As an alternative expression strategy, codon-optimised versions of the chimeric E559 heavy and light chain genes were cloned into expression cassettes arranged in tandem (head-to-tail orientation) in plant transformation vector pTRAk.2. Co-cultivation of *N. tabacum* leaf discs with an *A. tumefaciens* strain harboring the recombinant pTRAk.2 was used to generate transgenic tobacco lines, pTRAk-χE559, which were analyzed by ELISA for antibody assembly and yield (Figure 3*C*). The best yield of plant-derived chimeric E559 (χE559^P^) was determined to be 280 mg/kg of fresh leaf weight, approximately 150-fold greater than the nonoptimized, chimeric antibody expressed using the pL32 vector.

**Figure 4.**
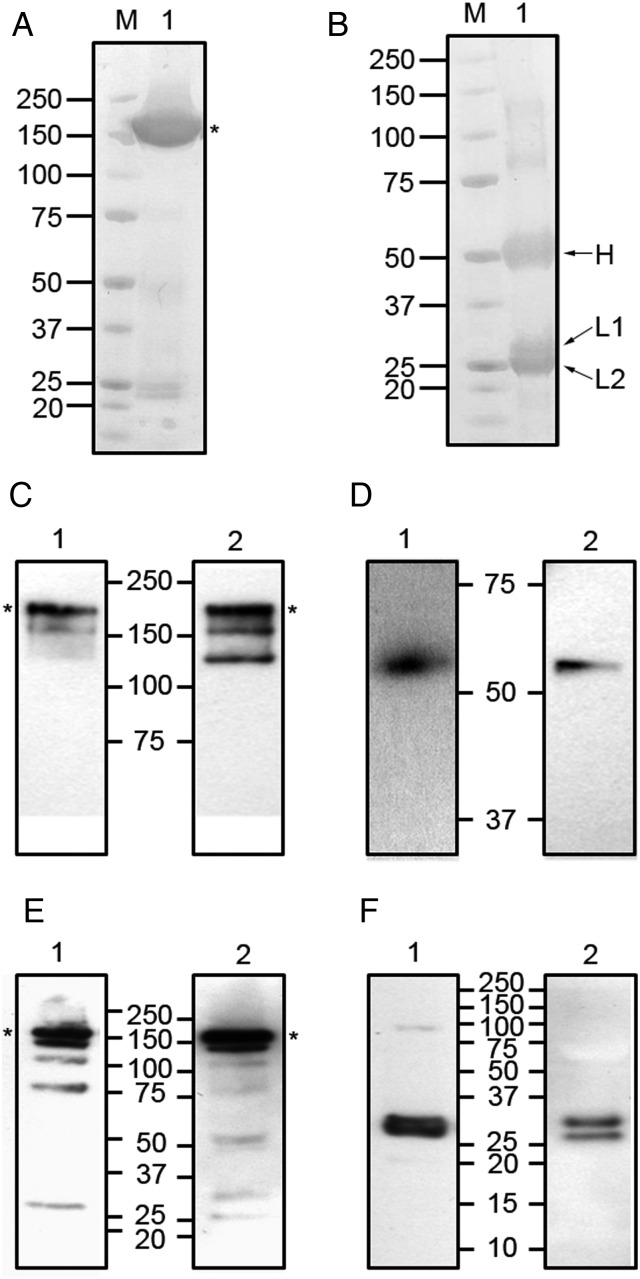
Gel and Western blotting analysis of purified χE559^P^. Purified, plant-derived chimeric E559 (χE559^P^) was analyzed by Coomassie staining of polyacrylamide gels under nonreducing (panel *A*) and reducing (panel *B*) conditions, and by Western blotting under nonreducing (panels *C* and *E*) and reducing (panels *D* and *F*) conditions. For Western blotting, the nitrocellulose membranes were probed with HRP-conjugated antibodies specific for heavy chains (panels *C* and *D*), or with HRP-conjugated antibodies specific for light chains (panels *E* and *F*). The χE559^P^ samples (lane 1) were probed with human-specific reagents, whereas E559^Hyb^ samples (lane 2) were probed with murine-specific reagents. Abbreviations: HRP, horseradish peroxidase; M, molecular weight standards. Asterisks indicate the positions of the fully assembled antibodies.

The purified χE559^P^ was analyzed by Coomassie staining under nonreducing and reducing conditions. A nonreducing gel (Figure 4*A*) showed a predominant high molecular weight band (indicated by the asterisk) at the expected size for the fully assembled antibody and, despite the presence of some minor low molecular weight bands, indicates a high degree of purity was achieved using the single-step (protein A/G) purification. The reducing gel (Figure 4*B*) shows the heavy chain (indicated by H) migrating at the expected position. As previously observed for the hybridoma-derived E559 (Figure 2*A*), the plant-derived χE559^P^ also comprises 2 isoforms of the light chain (indicated by L1 and L2). Additional higher molecular weight species in the reducing gel most likely represent incompletely reduced antibody.

The purified χE559^P^ was also analyzed by Western blotting, alongside purified E559^Hyb^. Figure 4*C* shows the results of a nonreducing blot, detected with antisera specific for human (lane 1) or mouse (lane 2) heavy chains. Both χE559^P^ and E559^Hyb^ samples have a high molecular weight band migrating at the expected position for the fully assembled antibody (indicated by the asterisk), with some additional lower molecular weight bands, representing either assembly intermediates or proteolytic fragments [34]. Under reducing conditions (Figure 4*D*), both samples showed a single band at approximately 55 kDa, corresponding to the expected size for free heavy chains.

Blotting of the light chains under nonreducing conditions (Figure 4*E*) revealed the fully assembled antibody for both χE559^P^ (lane 1) and E559^Hyb^ (lane 2) as well as some additional lower molecular weight species. Under reducing conditions (Figure 4*F*), E559^Hyb^ (lane 2) showed 2 bands, corresponding to the 2 glycoform variants, and χE559^P^ (lane 1) also showed 2 light chain species. The lower band in χE559^P^ corresponded in size to the lower band in E559^Hyb^, indicating that this is also an aglycosylated form of the light chain. The higher band in χE559^P^ had a slightly faster mobility compared to the glycosylated isoform of E559^Hyb^, and this probably reflects differences in the N-linked glycan structures between plants and mammals.

### Glycoproteomic Analysis

**Figure 5.**
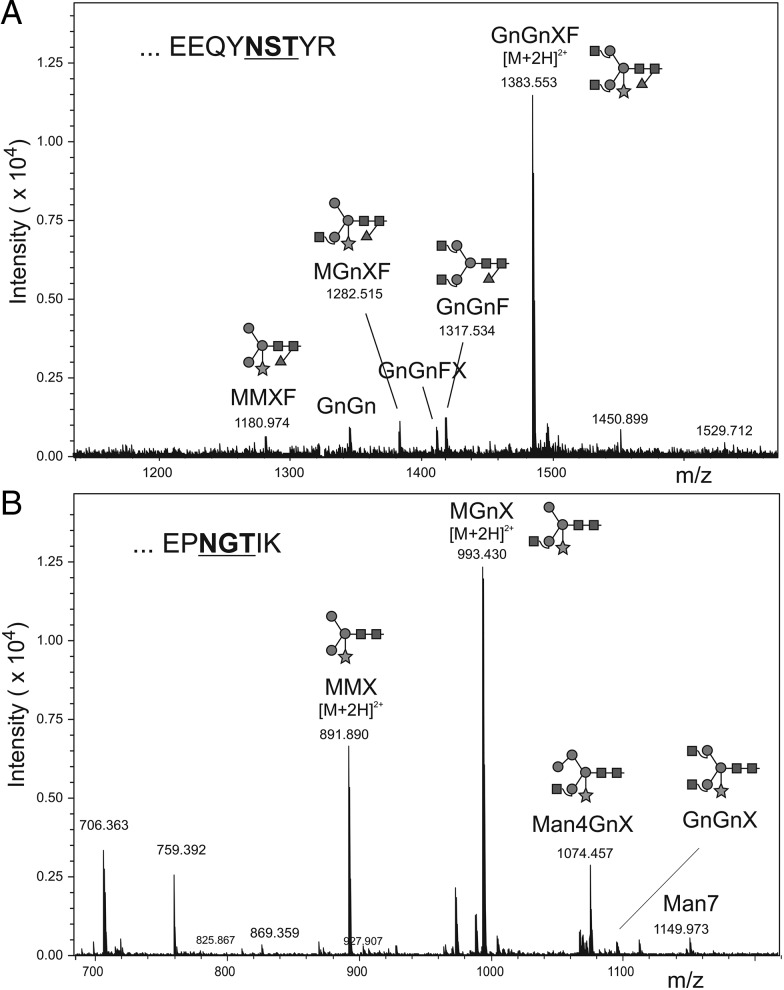
Glycan analysis of χE559^P^. Purified, plant-derived chimeric E559 was analyzed by in-gel digestion of *S*-carbamidomethylated sample and RP-ESI-MS. Deconvoluted spectra of the glycopeptide elution region of the Fc glycopeptide (*A*) and light chain glycopeptide (*B*). Masses correspond to oligomannosidic and complex-type structures. Abbreviations: F, fucose; Gn, *N*-acetylglucosamine; M, mannose; RP-ESI-MS, reverse-phase electrospray ionization mass spectrometry; X, xylose.

Sequence analysis of heavy and light chains of mAb E559 predicted the presence of 2 potential *N*-linked glycosylation sites, a conserved site in the antibody Fc region, and one in the V_L_ domain. The plant-derived antibody was subjected to glycoproteomic analysis by RP-ESI-MS (Figure 5). Glycopeptides comprising the Fc glycosylation site EEQFNSTFR (Figure 5*A*) and the V_L_ glycosylation site EPNGTIK (Figure 5*B*) were identified (N-linked glycosylation sites are underlined). The glycan analysis revealed that χE559^P^ heavy chain displayed glycan compositions typical of plant glycoproteins, with predominantly complex type glycans containing xylose and fucose (GnGnXF), which are presumed to be the β1,2-linked xylose residues attached to the β-linked mannose and the α1,3-fucose residue linked to the Asn-linked *N*-acetyl-glucosamine. The light chain glycosylation pattern (MGnX and MMX) was also largely typical of plant glycoproteins, except for the lack of the α1,3-fucose residue linked to the Asn-linked *N*-acetyl-glucosamine. Tandem MS results were subjected to Mascot MS/MS ion search, which confirmed the sample to contain essentially mAb E559.

### In vitro Neutralization

The hybridoma-derived E559 (E559^Hyb^) and both plant-derived antibodies (muE559^P^ and χE559^P^) were tested for neutralization of a diverse panel of lyssavirus species and strains using the mFAVN assay. The results (Table 1) show that both plant-derived antibodies mirrored the hybridoma-derived antibody in terms of breadth of neutralization. Representative viruses from phylogroups I and II [5, 40] were assayed for their ability to be neutralized by the antibodies. All tested phylogroup I viruses, covering the type species member (classical RABV), *Duvenhage virus*, *European bat lyssavirus* types 1 and 2, and *Australian bat lyssavirus*, were neutralized by all 3 antibodies and, except for *Duvenhage virus*, also by the OIE+ control. No neutralization was observed for the phylogroup II viruses tested (*Lagos bat virus* and *Mokola virus*).

**Table 1.** Virus Neutralizing Activity of Plant-derived Antibodies

Phylogroup	Lyssavirus Species (Genotype)	Virus Reference No.	Animal of Origin	Country of Origin	OIE+	muE559^P^	χE559^P^	E559^Hyb^
I	RABV (1)	CVS	Standard stock	n/a	+	+	+	+
		RV51	Fox	USA	+	+	+	+
		RV61	Human ex dog	UK (ex India)	+	+	+	+
		RV108	Bat	Chile	+	+	+	+
		RV410	Mongoose	South Africa	+	+	+	+
		RV437	Raccoon	Estonia	+	+	+	+
		RV1237	Deer	Yugoslavia	+	+	+	+
II	LBV (2)	RV1	Bat (*E. helvum*)	Nigeria	−	−	−	−
	MOK (3)	RV4	Shrew (*Crocidura spp.*)	Nigeria	−	−	−	−
I	DUVV (4)	RV131	Bat (*N. thebaica*)	Zimbabwe	−	+	+	+
	EBLV1 (5)	RV9	Bat (*E. serotinus*)	Germany	+	+	+	+
	EBLV2 (6)	RV1781	Bat (*M. daubentonii*)	UK	+	+	+	+
	ABLV (7)	RV634	Fruit bat	Australia	+	+	+	+

A modified fluorescent antibody virus neutralization (mFAVN) assay was used to compare the virus neutralizing activity of plant-derived chimeric E559 (χE559^P^), plant-derived murine E559 (muE559^P^), hybridoma-derived murine E559 (E559^Hyb^) and pooled dog reference sera from immunized animals (OIE+) against different lyssaviruses. Virus abbreviations: ABLV, *Australian bat lyssavirus*; CVS, challenge virus standard; DUVV, *Duvenhage virus*; EBLV1, *European bat lyssavirus* type 1; EBLV2, *European bat lyssavirus* type 2; LBV, *Lagos bat virus*; MOK, *Mokola virus*. Virus was considered neutralized if the neutralization titer was >0.5 IU/mL [36]. (+) indicates neutralization, (−) indicates no neutralization.

### In vivo Challenge Studies

The efficacy of the χE559^P^ in post-exposure prophylaxis was examined in hamsters injected with a lethal dose of a laboratory strain of RABV (CVS-11; Table 2). In this in vivo protection assay, all uninfected hamsters (group 1) survived. All hamsters that were infected with challenge virus and received mock PEP in the form of PBS (group 2) died after 14 days. The survival rates for hamsters that received PEP in the form of 22.5 IU/kg of either HRIG (group 3) or χE559^P^ (group 4) was >50% after 14 days, although after 28 days survival dropped to zero and 11% for HRIG and χE559^P^ groups, respectively. None of the groups received vaccine as part of the PEP regimen. The data show that the χE559^P^ antibody is at least as effective as the HRIG.

**Table 2.** In vivo Efficacy of χE559^P^ for Postexposure Prophylaxis

Group (Treatment)	14 d	28 d
Group 1 (Uninfected control)	4/4	4/4
Group 2 (PBS)	0/4	0/4
Group 3 (HRIG)	5/9	0/9
Group 4 (χE559^P^)	6/9	1/9

Four groups of Syrian hamsters were included in the experiment. Group 1 (uninfected control) animals did not receive any viral inoculum or biologics treatment. Groups 2, 3 and 4 were all inoculated with a genotype 1 RABV variant (at day 0) and treated subsequently (at day 1) with either PBS (group 2), HRIG (Rabigam) (group 3), or purified χE559^P^ mAb (group 4). Data are presented as the no. of surviving hamsters/no. of hamsters tested. Abbreviations: HRIG, human rabies immunoglobulin; PBS, phosphate-buffered saline.

## DISCUSSION

Current PEP for bites by rabid animals involves the use of blood-derived RIG, which can display batch-to-batch variation and may be of limited availability in case of sudden mass exposures. The concerns arising from the use of blood-derived products could be circumvented, and consistent batches of neutralizing antibodies could be produced in large quantities by adopting an approach based on a cocktail of rabies neutralizing mAbs. To this end, it is envisaged that RIG could be replaced by a mAb cocktail, produced using plants as the expression platform. Two different mAb production platforms in plants have already gained regulatory approval for human trials (Pharma-Planta Consortium, personal communication to J. Ma; [41]), demonstrating that plants are amenable to current Good Manufacturing Practice (cGMP) compliance [42].

We compared the murine hybridoma-derived E559 (E559^Hyb^) with the same murine antibody produced in *N. tabacum* (muE559^P^), as well as a mouse-human chimeric version (χE559^P^), also expressed in *N. tabacum*. In vitro testing of virus neutralization demonstrated that all 3 versions of E559 were equivalent, with all 3 neutralizing phylogroup I viruses but not the phylogroup II viruses. This is in accord with previous reports showing that neutralizing antibodies targeting phylogroup I viruses are not effective at neutralizing phylogroup II viruses [40, 43].

E559 has a predicted glycosylation site in the framework region of the V_L_ domain, which appears to be utilized, as 2 forms of the hybridoma-derived light chain (glycosylated and aglycosylated) are observed under reducing conditions, with the higher molecular weight form disappearing after treatment with PNGaseF. Two isoforms were also observed in the plant expressed χE559^P^. The effect of the V_L_ glycosylation is unknown, as both glycosylated and aglycosylated forms of the light chain were present in the hybridoma and plant preparations used for assessment of antibody functionality.

Purified χE559^P^ was analyzed by mass spectrometry and was shown to be glycosylated with typical plant complex glycan structures. It is well established that plant N-linked glycosylation differs from murine glycosylation [44], due to differences in complex glycan processing in the Golgi compartment. Previous studies have shown that plant-derived mAbs can have different half-lives in animals, compared to mammalian-derived mAbs [25, 45]. Although these differences have been attributed to differences in glycosylation, a more recent study [46] found no difference in the clearance rates of a RABV-neutralizing human mAb expressed in hybridoma cells or plants. The impact on the in vivo half-life of the glycosylation differences between E559^Hyb^ and χE559^P^ will need to be addressed in human trials.

Functionally, χE559^P^ retained neutralization activity and had the same breadth of lyssavirus coverage as E559^Hyb^. In vivo, the chimeric antibody was as effective as a commercial HRIG product in a hamster challenge model.

The potential for viral escape, and the need to provide protection across a broad range of lyssaviruses, means that a single mAb will probably not be sufficient for a rabies PEP product, and this has been recognized by various groups [15, 17, 18, and 31]. However, the cost of mAbs produced in mammalian cell bioreactors is currently prohibitive for rabies products intended for use in resource-poor settings, so it seems unlikely that products combining 2 or more mAbs produced using such traditional platforms will be commercially viable outside the developed world. Production of RABV-neutralizing mAbs in plants raises hopes that these mAbs will be available in quantities sufficient to meet the needs for PEP in rabies-endemic areas, particularly across the developing world.

## Supplementary Data

Supplementary materials are available at *The Journal of Infectious Diseases* online (http://jid.oxfordjournals.org/). Supplementary materials consist of data provided by the author that are published to benefit the reader. The posted materials are not copyedited. The contents of all supplementary data are the sole responsibility of the authors. Questions or messages regarding errors should be addressed to the author.

Supplementary Data
