# Supplementary material for: Engineering, Expression in Transgenic Plants and Characterisation of E559, a Rabies Virus-Neutralising Monoclonal Antibody
Source: J Infect Dis. 2014 Feb 7;210(2):200–8. doi: 10.1093/infdis/jiu085 (PMC4073784; doi:10.1093/infdis/jiu085)
Supplement: Supplementary Data [file supp_210_2_200__index.html]

Engineering, Expression in Transgenic Plants and Characterisation of E559, a Rabies Virus-Neutralising Monoclonal Antibody — Engineering, Expression in Transgenic Plants and Characterisation of E559, a Rabies Virus-Neutralising Monoclonal Antibody — Supplementary Data 

# Engineering, Expression in Transgenic Plants and Characterisation of E559, a Rabies Virus-Neutralising Monoclonal Antibody

## Supplementary Data

Supplementary Data

**Files in this Data Supplement:**

- Supplementary Data - Docx file
