# Supplementary material for: Engineering, Expression in Transgenic Plants and Characterisation of E559, a Rabies Virus-Neutralising Monoclonal Antibody
Source: J Infect Dis. 2014 Feb 7;210(2):200–8. doi: 10.1093/infdis/jiu085 (PMC4073784; doi:10.1093/infdis/jiu085)
Supplement: Supplementary Data [file supp_jiu085_jiu085supp.docx]

**Supplementary Information for van Dolleweerd et al., Engineering, expression in transgenic plants and characterisation of E559, a rabies virus-neutralising monoclonal antibody**

**Supplementary Material and Methods**

***Plant transformation vectors***

Plant transformation vector pL32 was used for cloning and expression of the murine and chimeric (mouse-human) forms of E559. pL32 is a derivative of the binary vector pMON530 [[1](#_ENREF_1)], modified by insertion of the coding sequence for a murine immunoglobulin heavy chain leader sequence, so that genes of interest fused downstream of the leader are co-translationally translocated into the plant secretory pathway/endomembrane system. Gene expression is under the control of the promoter for the cauliflower mosaic virus 35S transcript (CaMV-35S) and the nopaline synthetase (nos) 3’ terminator. For expression of a codon-optimised version of the chimeric mouse-human E559, plant transformation vector pTRAk.2 was used. Plasmid pTRAk.2 is a derivative of pTRAk [[2](#_ENREF_2)], where gene expression is under the control of a duplicated CaMV-35S promoter, a 5’-untranslated region from tobacco etch virus, a CaMV-35S transcriptional terminator and scaffold attachment regions of the tobacco *RB7* gene. The T-DNA regions of both pL32 and pTRAk.2 harbour the *neomycin phosphotransferase II* gene, which permits kanamycin selection of transformed plants.

***Cloning of chimeric mouse-human E559 IgG***

For production of the chimeric mouse-human heavy chain (χE559H), the variable domain (V_H_) of the E559 heavy chain was amplified using primers FR1γ and E559H#8. A heavy chain constant region (C_H_) was amplified from the human γ1 heavy chain gene from subclass-switched monoclonal antibody 4E10 (see [[3](#_ENREF_3), [4](#_ENREF_4)]) using primers 4E10H#3 and 4E10H#4. The variable and constant domains were fused together using splicing-by-overlap extension [[5](#_ENREF_5)], digested with *Xho*I and *Eco*RI and cloned into pL32 for expression in plants. The same method was used for construction of the chimeric light chain (χE559L), using forward primer FR1κ and reverse primer E559L#5 to amplify the E559 V_L_ domain, and forward primer 4E10L#1 together with reverse primer 4E10L#2 to amplify the constant region from the 4E10 kappa light chain gene.

***Codon-optimised, chimeric mouse-human E559 IgG***

For the codon-optimised version of the chimeric mouse-human E559, the codon usage of the chimeric heavy and light chain genes was optimised for *Nicotiana tabacum* expression using the codon optimisation service from GeneArt (Invitrogen). Codon-optimised genes were cloned as tandem expression cassettes, arranged head-to-tail, in the plant transformation vector pTRAk.2 for coexpression in transgenic plants. Human immunoglobulin leader sequences were used to target the immunoglobulin chains into the secretory pathway.

***Generation and screening of transgenic Nicotiana tabacum plants***

Tobacco leaf discs from *Nicotiana tabacum* cv. Petite Havana SR1 were transformed by co-cultivation either with *Agrobacterium tumefaciens* strain LBA4404 harbouring recombinant pL32 plasmids, or with *A. tumefaciens* strain GV3101::pMP90RK harbouring recombinant pTRAk.2 plasmid. Leaf discs were cultured on Murashige and Skoog Basal Medium, pH 5.8 (Sigma, UK) containing 3% (w/v) sucrose, 1 μg/mL Benzylaminopurine, 0.1 μg/mL α‑Naphthaleneacetic acid, 0.8% (w/v) Bacto™ agar, and 200 μg/mL kanamycin to select for transformants. After shoot formation, induction of roots was achieved by transferring to Murashige and Skoog Basal Medium, pH 5.8 (Sigma, UK) containing 3% (w/v) sucrose, 0.8% (w/v) Bacto™ agar and 200 μg/mL kanamycin. Transgenic plants were maintained in soil. Plants were sexually crossed by transferring the pollen from one flower onto the stigma of another flower, from which the anthers had been removed (to prevent self-crossing).

***Mass spectrometry analysis of hybridoma-derived E559 (E559^Hyb^) light chain***

Bands of interest were excised from Coomassie-stained acrylamide gels and washed with UCL/MS grade water (Biosolve). Unbound Coomassie stain was removed by washing with 50 mM NH_4_HCO_3_ in 50% (v/v) acetonitrile. Protein bands were reduced using 10 mM dithiothreitol in 100 mM NH_4_HCO_3_ and alkylated using 55 mM iodoacetic acid in 100 mM NH_4_HCO_3_. Gel pieces were dehydrated with 100% acetonitrile and dried using a SpeedVac concentrator (Thermo Scientific). Trypsin (Promega sequencing grade, resuspended to 100 ng/μL with 0.1% (v/v) trifluoroacetic acid) was added to the dried gel pieces and incubated for 16 hours at 37°C. Released peptides were retrieved by washing three times with 50% (v/v) acetonitrile and 0.1% (v/v) trifluoroacetic acid and once with 100% acetonitrile. Peptides were analysed by LC-MS/MS using a Surveyor LC system and LCQ Deca XP Plus mass spectrometer (ThermoScientific). Briefly, peptides were resolved by reverse phase chromatography (Biobasic column, ThermoScientific; 180 μm x 15 mm) over a 30 minute acetonitrile gradient at a flow rate of 2 μL/min. Peptides were ionised by electrospray ionisation (Ion Max source, 34-gauge stainless steel needle fitted in the ESI probe, ThermoScientific) and MS/MS was acquired on selected ions dependant on their charge state and intensity. Mass accuracy and sensitivity of the MS was confirmed with the direct infusion of glufibrinopeptide (2.5 pmol/μL) and LC-MS/MS performance was assessed with a digest of bovine serum albumin (BSA). Sensitivity, retention time, peptides identified and protein sequence coverage were all within the specified ranges. BSA quality control checks were performed prior to the analysis of the sample and post-acquisition.

***Deglycosylation using PNGaseF***

Protein G-purified E559^Hyb^ was dialysed into 50 mM sodium phosphate buffer, pH 7.5 and 5 μg of the dialysed antibody (in 50 μL) was treated with 5 μL of 0.2% (w/v) sodium dodecylsulfate, 100 mM 2‑mercaptoethanol at 100^o^C for 10 minutes to denature the mAb. After cooling, 5 μL of triton X-100 was added. A 10 μL aliquot of the PNGaseF stock (lyophilised Peptide-N-glycosidase F (PNGaseF) from *E. miricola* (Sigma, UK) resuspended to 500 U/mL) was added to the denatured mAb and the mixture was incubated at 37^o^C for 12 hours. The reaction was stopped by heating to 100^o^C for 5 minutes.

***Enzyme-linked immunosorbent assay (ELISA)***

For detection of antibody heavy or light chains, 96-well Maxisorp, flat-bottom ELISA plates (NUNC, Denmark) were coated with 50 μL/well of either sheep anti-mouse IgG1 (The Binding Site), goat anti-mouse IgG, light chain-specific (Jackson ImmunoResearch, Stratech, UK), sheep anti-human IgG1 (The Binding Site), or sheep anti-human kappa (The Binding Site) antisera, diluted 1/500 in PBS. Plates were incubated at 37^o^C for 2 hours, washed twice with distilled water and free protein binding sites were blocked by incubating overnight at 4^o^C with 200 μL/well of Blocking Buffer (5% (w/v) non-fat milk powder in PBS). After washing in Wash Solution (0.1% (v/v) Tween-20 in deionised water), samples were added to the wells and serially diluted in Dilution Buffer (5% (w/v) non-fat milk powder, 0.1% (v/v) Tween-20 in PBS). Commercially available, isotype-matched human IgG1κ (The Binding Site) or mouse IgG1κ (Sigma, UK) antibodies were used as positive controls and to generate a standard concentration curve. Plates were incubated at room temperature for 2 hours and washed six times in Wash Solution. Captured immunoglobulin chains were detected by the addition of 50 μL/well of horseradish peroxidase (HRP)-labelled antibodies (diluted 1/1000 in Dilution Buffer) and incubated at room temperature for 2 hours. Following six washes in Wash Solution, 50 μL/well of TMB substrate solution (3,3’,5,5’-tetramethylbenzidine (Sigma, UK) in 0.025 M citrate-0.05 M phosphate buffer, pH 5.0) was added. The colour reaction was stopped by addition of 25 μL/well of 2 M sulphuric acid, and the absorbance was determined at 450 nm using a Sunrise plate reader (Tecan, UK).

***SDS-PAGE and western blotting***

Protein samples were separated on 4-12% gradient polyacrylamide minigels (Invitrogen) using NuPAGE buffers (Invitrogen). Following electrophoresis, gels were stained with Coomassie Brilliant Blue or proteins were blotted onto nitrocellulose membranes using a semi-dry transfer device (BioRad). Following transfer, membranes were incubated in Blocking Buffer for at least 30 minutes to block free protein binding sites. A polyclonal HRP-labelled sheep anti-mouse IgG1 reagent (The Binding Site) was used for detection of murine heavy chains. Detection of murine light chains was performed with an HRP-labelled goat anti-mouse IgG, light chain-specific antibody (Jackson ImmunoResearch, Stratech, UK). A polyclonal HRP-conjugated sheep anti-human IgG1 reagent (The Binding Site) was used for detection of chimeric mouse-human heavy chains, and HRP-labelled sheep anti-human kappa light chain reagent (The Binding Site) for detection of chimeric mouse-human light chains. Antisera were diluted 1/2000 in Dilution Buffer. Blots were washed five times with Wash Solution and developed using the ECL Plus western blotting detection system (GE Healthcare).

***Modified fluorescent antibody virus neutralisation (mFAVN) assay***

Monoclonal antibodies were added to cell culture media (DMEM media supplemented with 10% fetal calf serum and 1% Penicillin/Streptomycin) and titrated in 4-fold dilutions across a 96-well plate, starting at 1/8 dilution. Controls were “cells alone”, “virus alone” and “cells and virus” which were set up with the appropriate volume of media. The mAbs and live viruses were incubated at 37^o^C (5% CO_2_) for 1 hour before adding 4x10^5^ baby hamster kidney (BHK) cells to each well. The plates were incubated at 37^o^C (5% CO_2_) for 48 hours, washed three times in PBS (pH 7.2), fixed in 80% acetone and air-dried. The staining was carried out by addition of 50 μL of fluorescein isothiocyanate (FITC)-conjugated antibody specific for RABV nucleoprotein (Centocor) to each well. Plates were incubated at 37^o^C (5% CO_2_) for 30 minutes, washed three times with PBS (pH 7.2) and evaluated by fluorescent microscopy. Pooled dog sera from vaccinated animals (OIE positive, OIE+) and OIE negative (OIE-) reference sera were included as controls. Virus was considered neutralised if the neutralisation titre was >0.5 IU/mL [[6](#_ENREF_6)].

**Supplementary Table S1. Oligonucleotide primers used for cloning.** Primers used for isolation and cloning of the murine heavy and light chain genes from hybridoma E559.9.14, and for cloning the chimeric mouse-human genes are shown. Single letter nucleotide codes are as defined by IUPAC (<http://www.chem.qmul.ac.uk/iupac>).

| **Primer Name** | **Primer Sequence** | **Features** |
| --- | --- | --- |
| FR1γ | 5’‑tggtacctcgagc**caggtsmarctgcagsagtcwg**‑3’ | Degenerate forward primer for amplifying murine γ1 heavy chain genes. Bold sequence corresponds to the start of the V_H_ domain. *Xho*I site underlined. |
| 932 | 5’-cctgtaggaccagaggaattcgtcgacactgggatta**tttac**-3’ | Reverse primer for amplifying murine γ1 heavy chain genes. Bold sequence corresponds to the end of the C_H_3 domain. *Eco*RI site underlined. |
| FR1κ | 5’‑gtggtacctcgagc**gayatyswgmtsacccartct**‑3’ | Degenerate forward primer for amplifying murine κ light chain genes. Bold sequence corresponds to the start of the V_L_ domain. *Xho*I site underlined. |
| 933 | 5’‑ggggagctggtggtgaattcgtcgacctttgtctcta**acactc**‑3’ | Reverse primer for amplifying murine κ light chain genes. Bold sequence corresponds to the end of the C_κ_ domain. *Eco*RI site underlined. |
| E559H#8 | 5’‑cgatgggcccttggtggaagc**tgaggagactgtgagagtgga**‑3’ | Reverse primer for amplifying the V_H_ domain of E559. Bold sequence correspond to the 3’ end of the E559 V_H_ domain. Underlined sequence corresponds to the 5’ end of the 4E10 C_H_1 domain. |
| 4E10H#3 | 5’‑tccactctcacagtctcctca**gcttccaccaagggcccatcg**‑3’ | Forward primer for amplifying the constant region from the γ1 heavy chain gene of 4E10. Bold sequence correspond to the start of the 4E10 C_H_1 domain. Underlined sequence corresponds to the 3’ end of the E559 V_H_ domain. |
| 4E10H#4 | 5’‑gtcggaattcgcggccgctca**tttacccggagacaggga**-3’ | Reverse primer for amplifying the γ1 heavy chain gene from human mAb 4E10. Bold sequence corresponds to the end of the 4E10 C_H_3 domain. *Eco*RI site underlined. |
| E559L#5 | 5’‑agatggtgcagccacagttcg**ttttatttcaagcttggtccc**‑3’ | Reverse primer for amplifying the V_L_ domain of E559. Bold sequence correspond to the 3’ end of the E559 V_L_ domain. Underlined sequence corresponds to the 5’ end of the 4E10 C_κ_ domain. |
| 4E10L#1 | 5’‑gggaccaagcttgaaataaaa**cgaactgtggctgcaccatct**‑3’ | Forward primer for amplifying the constant region from the κ light chain gene of 4E10. Bold sequence correspond to the start of the 4E10 C_κ_ domain. Underlined sequence corresponds to the 5’ end of the 4E10 C_κ_ domain. |
| 4E10L#2 | 5’‑tctagaattcgcggccgccta**acactctcccctgttgaa**‑3’ | Reverse primer for amplifying the κ light chain gene from human mAb 4E10. Bold sequence corresponds to the end of the 4E10 C_κ_ domain. *Eco*RI site underlined. |

**Supplementary References**

**1. Rogers SG, Klee HJ, Horsch RB, Fraley RT. Improved Vectors for Plant Transformation: Expression Cassette Vectors and New Selectable Markers. Methods in Enzymology 1987; 153:253-77.**

**2. Sack M, Paetz A, Kunert R, et al. Functional analysis of the broadly neutralizing human anti-HIV-1 antibody 2F5 produced in transgenic BY-2 suspension cultures. FASEB J 2007; 21:1655-64.**

**3. Stiegler G, Kunert R, Purtscher M, et al. A potent cross-clade neutralizing human monoclonal antibody against a novel epitope on gp41 of human immunodeficiency virus type 1. AIDS Res Hum Retroviruses 2001; 17:1757-65.**

**4. Kunert R, Steinfellner W, Purtscher M, Assadian A, Katinger H. Stable recombinant expression of the anti HIV-1 monoclonal antibody 2F5 after IgG3/IgG1 subclass switch in CHO cells. Biotechnol Bioeng 2000; 67:97-103.**

**5. Horton RM, Hunt HD, Ho SN, Pullen JK, Pease LR. Engineering hybrid genes without the use of restriction enzymes: gene splicing by overlap extension. Gene 1989; 77:61-8.**

**6. Cliquet F, Aubert M, Sagne L. Development of a fluorescent antibody virus neutralisation test (FAVN test) for the quantitation of rabies-neutralising antibody. J Immunol Methods 1998; 212:79-87.**
